# A synthetic method to assay polycystin channel biophysics

**DOI:** 10.1101/2024.05.06.592666

**Published:** 2024-05-06

**Authors:** Megan Larmore, Orhi Esarte Palomero, Neha P. Kamat, Paul G. DeCaen

**Affiliations:** 1. Department of Pharmacology, Feinberg School of Medicine, Northwestern University, Chicago, Illinois, USA.; 2. Department of Biomedical Engineering, McCormick School of Engineering and Applied Science, Northwestern University, Evanston, Illinois, USA.; 3. Center for Synthetic Biology, Northwestern University, Evanston, Illinois, USA; 4. Chemistry of Life Processes Institute, Northwestern University, Evanston, Illinois, USA

## Abstract

Ion channels are biological transistors that control ionic flux across cell membranes to regulate electrical transmission and signal transduction. They are found in all biological membranes and their conductive states are frequently disrupted in human diseases. Organelle ion channels are among the most resistant to functional and pharmacological interrogation. Traditional channel protein reconstitution methods rely upon exogenous expression and/or purification from endogenous cellular sources which are frequently contaminated by resident ionophores. Here we describe a fully synthetic method to assay the functional properties of the polycystin subfamily of transient receptor potential (TRP) channels that natively traffic to primary cilia and endoplasmic reticulum organelles. Using this method, we characterize their membrane integration, orientation and conductance while comparing these results to their endogenous channel properties. Outcomes define a novel synthetic approach that can be applied broadly to investigate other channels resistant to biophysical analysis and pharmacological characterization.

## INTRODUCTION

Ion channels are pore forming integral transmembrane proteins essential for all cellular lifeforms^1, 2^. At the plasma membrane, ion channels are responsible for generating long range bioelectric conduction in excitable eukaryotic cells (e.g. neurons), and within prokaryotic colonies (e.g. bacteria biofilms), and filamentous colonies of archaea^3–5^. Here, they contribute to fundamental vital cell processes including division, signal transduction, and ionic homeostasis^1^. In eukaryotic organelle membranes, ion channels are integral to a wide range of functions including energy production (mitochondria), and the maintenance of defining compartmental conditions such as Ca^2+^ gradients (endoplasmic reticulum), pH (lysosome) and redox states (peroxisome)^6–8^. The conductive states of channels are precisely controlled by their molecular conformations which are unique among their phylogenetic families and subfamilies^9^. More than 400 human genes encode ion channel subunits, many of which are altered by variants that impact organ function and development^10–12^. These so called ‘Channelopathies’ manifest in various human diseases such as cardiac arrhythmias, neurological conditions, and cystic kidney diseases^13, 14^. Besides their association with disease-causing variants, channels are important therapeutic targets for treating various pathophysiologies and represent the second largest target among the existing FDA-approved drug portfolio^15–17^. However, many members of the so called “dark genome” of understudied proteins encode putative ion channels that are implicated in human disease but remain resistant to functional characterization^18^. Furthermore, channels which localize to cellular compartments in low quantities present a significant challenge to assay for drug screening purposes^19^. Thus, these observations warrant the present investigation of a novel methodological approach to characterize ion channel biophysics and pharmacology.

Voltage clamp electrophysiology incarnated in either planar or glass electrode designs provide direct, real-time and the highest available fidelity measurements of the ion channel conductive states. In the on-cell or inside-out configurations, their opening and closing conformational changes (i.e. gating) are captured as stochastic single channel currents after forming high resistance seals (>10 MΩ) at the interface of the electrode against a biological membrane^20^. This conventional electrophysiology technique is commonly used to characterize the properties of plasma membrane channels and typically involves either recording from an endogenous cell source or from a cell line expressing the channel transgene^21, 22^. However, capturing the biophysical properties of organelle channels and those from bacteria can be mired by electrode inaccessibility to inner membranes. As a work around, investigators have employed various channel reconstitution methods which typically consist of several steps^23, 24^. First, the channel of interest is expressed and immunopurified from a biological cell source, then it is reconstituted into a synthetic or into biologically derived lipid bilayers or vesicles. While there are many examples where heterologous approaches have faithfully reproduced the functional properties of channels from native sources, these preparations are frequently contaminated by endogenous ionophores from the host cell lines, even after purification^25, 26^.

To address this, we have developed a completely synthetic method to assay ion channel biophysics. The approach combines advances in cell-free protein expression (CFE) and reconstitution of the synthetic channel protein into giant unilamellar vesicles (GUV) where their single channel properties can be measured using voltage-clamp electrophysiology^27–29^. CFE is a form of *in vitro* protein synthesis, utilizing purified cellular machinery (ribosomes, tRNAs, enzymes, cofactors, etc.) needed to directly transcribe and translate user-supplied DNA plasmid encoding an ion channel. The unmodified channel proteins are reconstituted into GUVs— cell-sized model membrane systems derived from electrolysis of synthetic lipid mixtures. To validate this method, we characterized PKD2 and PKD2L1 channels, two members of the polycystin subfamily of transient receptor potential (TRP) ion channels. Polycystin subunits can form hetero- and homotetrameric ion channel complexes with several members of the TRP family, where they traffic to the primary cilia and ER organelle membranes^30^. Both features present challenges for experimentalist to functionally characterize these channel properties within their endogenous cell membranes. The medical and biological importance of polycystins is underscored by PKD2 variants associated with autosomal dominant polycystic kidney disease (ADPKD), and this channel’s role in fertility and conferring right-left symmetry in embryonic development^31–33^. While PKD2L1 variants have yet to be linked to human disease, its loss of expression results in epilepsy susceptibility and autism -like features in mice^34, 35^. In this report, we stepwise express and confirm protein expression of polycystin channels using the CFE method; reconstitute channel protein in GUVs containing distinct lipid components; assess correct membrane orientation using self-labeling saturated N-heterocyclic building blocks (SNAP) protein chemistry and evaluate channel properties using electrophysiology^36^. Outcomes define a novel reductionist and generalizable approach to study ion channels resistant to biophysical characterization.

## RESULTS AND DISCUSSION

**Figure 1.**
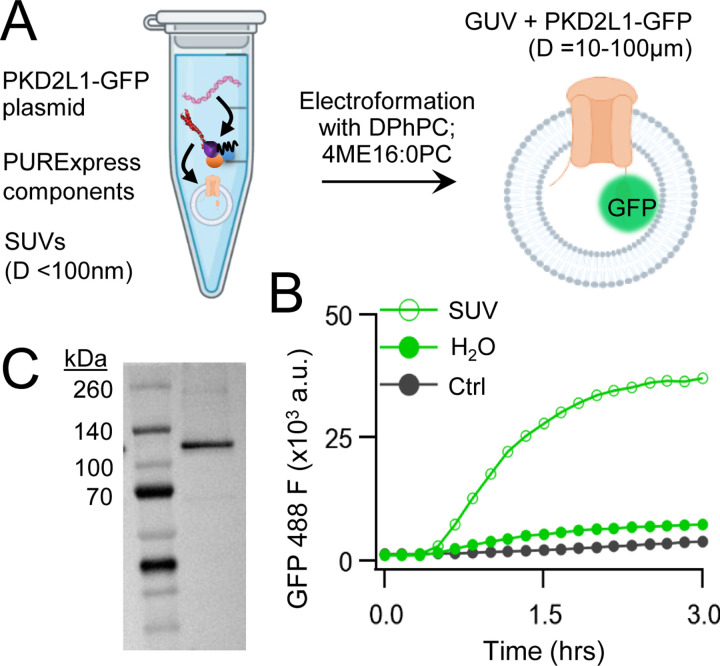
Cell-free expression of synthetic PKD2L1 protein and incorporation into lipid vesicles. **A**) Schematic of cell-free protein expression into synthetic lipid vesicles and subsequent electroformation with Vesicle Prep Pro (Nanion). **B)** Monitored fluorescence over time of cell-free expressed PKD2L1-GFP and a non-fluorescent control plasmid produced in the presence or absence of lipid vesicles. **C**) Full length PKD2L1-GFP protein detected by Western Blot after cell-free expression into vesicles.

We began by carrying out CFE in vitro synthesis of PKD2L1 protein in the presence and absence of lipid vesicles. Plasmid DNA encoding human PKD2L1 with C-terminally tagged green fluorescent protein (PKD2L1-GFP) was added to the cell-free expression components (PURExpress, New England Biolabs) and induced protein translation by heat (Figure 1A, See methods). Each reaction produced 8 ± 3.5 μg/μl of synthetic channel protein as estimated by a standardized GFP absorbance assay (Figure 1— figure supplement 1A). The synthetically derived channel protein identity was confirmed using two methods. First, by western blot of the cell-free reaction where the PKD2L1-GFP protein was SDS-PAGE gel separated from the reactants and detected by an anti-GFP monoclonal antibody (Figure 1B). Second, the PKD2L1 protein was confirmed by mass spectroscopy with 46% coverage (Figure 1— figure supplement 1B,C). Since polycystins are transmembrane proteins, we hypothesize that channel membrane incorporation would be facilitated by including lipid substrates into the CFE reaction. Thus, we compared channel synthesis in the presence or absence of small unilamellar vesicles (SUVs) comprised of 1,2-diphytanoyl-sn-glycero-phosphocholine (DPhPC; 4ME16:0PC) and cholesterol (Figure 1A). We monitored PKD2L1-GFP protein production over time through fluorescence which is dependent on complete channel translation and correct GFP folding^37^. We observed a dramatic increase in fluorescence output which plateaued after 3 hours when CFEs reactions were supplemented with SUVs—and affect not observed in water-supplement (H_2_O) control reactions (Figure 1C)^38^. Minimal changes in fluorescence detected when a control plasmid (Ctrl) encoding a non-fluorescent protein (dihydrofolate reductase) was used in the reaction. Taken together, these results demonstrate the efficacy of synthesizing full length polycystin channels using the cell free expression system, and the enhancement of transmembrane protein synthesis through lipid vesicle incorporation during these reactions.

**Figure 2.**
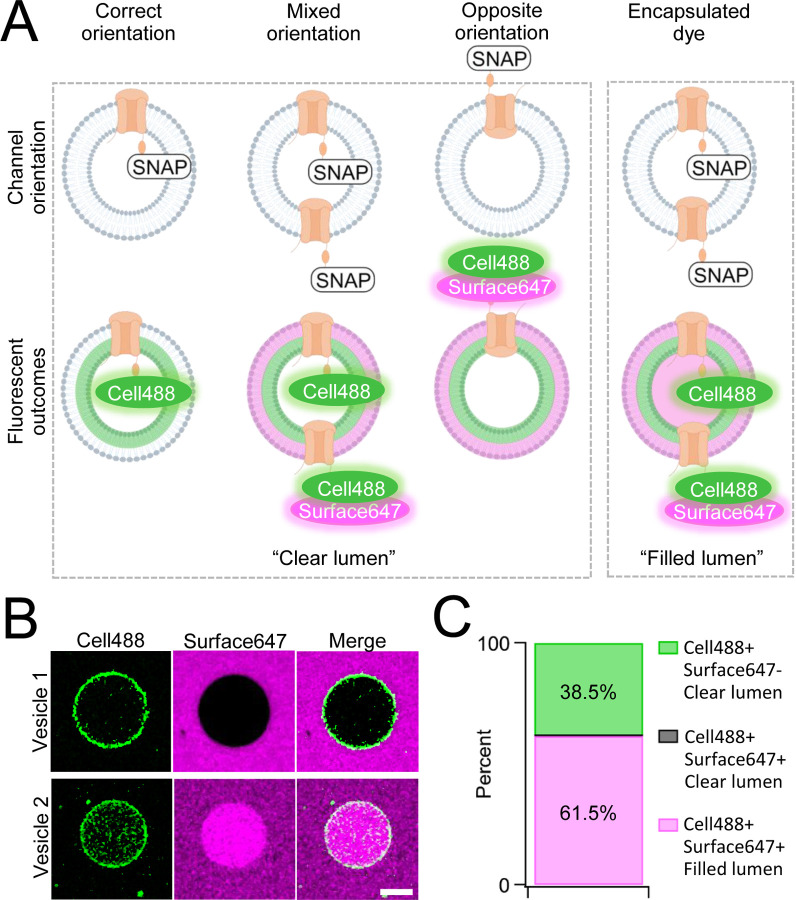
Orientation of synthetic PKD2L1 channels in vesicles. **A**) Schematic of possible ion channel orientation outcomes from PKD2L1 cell-free expression (top) and hypothesized fluorescence results when Cell488 and Surface647 added (bottom). **B**) Fluorescent confocal images from the SNAP-tagged vesicles. Scale bar represents 10 μm for all images. **C**) Vesicle percentage depicts percent of vesicles with each fluorescent output, (N=65).

One caveat of membrane protein vesicle reconstitution is the potential for misorientation after integration. To assay channel orientation, we synthesized PKD2L1 with a C-terminal SNAP-tag fusion protein (PKD2L1-SNAP). The SNAP-tag specifically reacts with fluorescent O2-benzylcytosine derivatives and, depending on the membrane permeability, will react with lipid integrated proteins based on the tag’s accessibility (Figure 2— figure supplement 1). GUVs containing CFE synthesized PKD2L1-SNAP were formed by electroformation after passing current through indium tin oxide slides treated with dried SUV-channel protein mixture^39^. We then added two substrates, membrane permeable SNAP-Cell Oregon Green (Cell488) and membrane impermeable SNAP-Surface Alexa Fluor 647 (Surface647), to monitor orientation-dependent membrane protein reactivity with the SNAP label (Figure 2A, Figure 2— figure supplement 1). We hypothesized there will be two fluorescent outcomes. First, if all channels orient correctly, then we should see only Cell488 fluorescence with no Surface647 at the membrane (Figure 2A). Second, if the channels are in opposite or in mixed orientations, then we expect to see dual fluorescence of Cell488 and Surface647 (Figure 2B). We imaged over 60 GUVs and found 38.5% of the vesicles exhibited sole Cell488 fluorescence— indicating their correct channel orientation within this population (Figure 2B, C). Importantly, none of the vesicle membranes labeled with both Cell488 and Surface647 while retaining a clear lumen— suggesting that the population of GUVs containing misoriented channels was nominal. In some cases, vesicles can encapsulate dye through compromised integrity or mechanisms other than membrane permeability. This is apparent when fluorescence can be seen in the vesicle lumen, rather than on the membrane (Figure 2C). While the encapsulated fluorescent vesicles account for most of the vesicles imaged, there were no vesicles seen with Cell488 and Surface647 fluorescence at the membrane with a clear lumen (Figure 2B, C). Based on these results, we conclude that our cell-free synthesized PKD2L1 channels are successfully reconstituted in GUVs in the correct orientation, and this preparation is suitable assaying PKD2L1 channels using electrophysiology.

**Figure 3.**
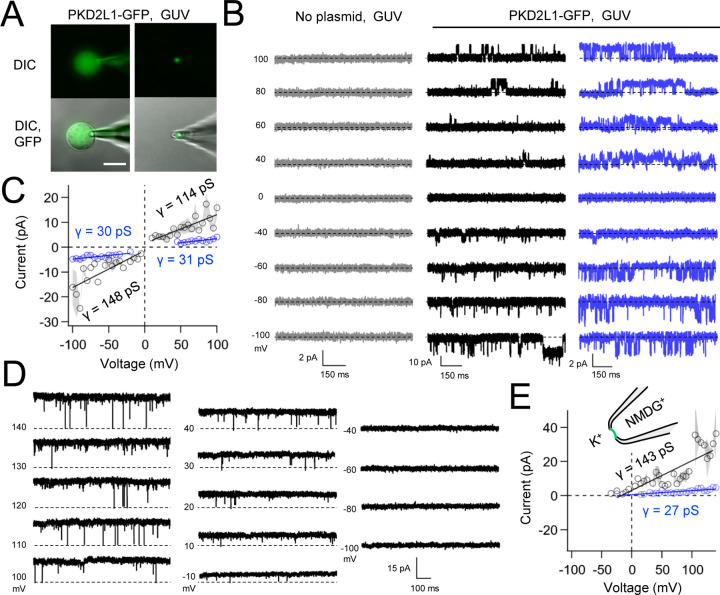
Synthetic PKD2L1 channels are functional in artificial membranes. **A**) Images of voltage clamped GUVs with incorporated PKD2L1-GFP channels. *Left*, establishing high-resistance seals in the on-cell patch configuration. *Right*, transitioning to the inside-our patch configuration. Scale bar = 20 μm. **B**) Example unitary single channel current records from GUVs reconstituted with or without PKD2L1 protein. Vesicles were patched using the symmetrical 150 mM K^+^ conditions (see methods) and PKD2L1 single channel current producing full and sub-conductances are colored black and blue, respectively. **C**) Average single-channel current amplitudes. Conductance (γ) estimated by fitting the average single channel currents to a linear equation. Error (grey) indicate SEM from N=2–8 GUVs. Several replicates lacked single channel openings at all test potentials. Conductance estimates from individual GUVs are shown in Figure 3—Supplement Figure 1A. **D**) PKD2L1 single channel current recorded using asymmetric cationic solutions, with 150 mM K^+^ in the bath and 150 mM NMDG^+^ in the pipette. **E**) Resulting average single-channel current amplitudes where no inward single channel current was detected (N=1–4 GUVs).

**Figure 4.**
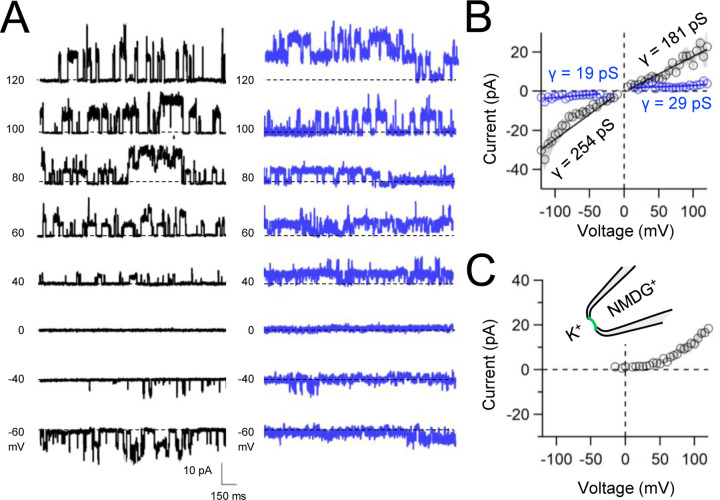
Functional synthetic PKD2 channels in artificial membranes. **A)** Example unitary single channel current records from GUVs reconstituted with PKD2 protein in symmetrical 150 mM K^+^ conditions producing full (black traces) and sub-conductances (blue traces), respectively. **B**, **C**) Average single-channel current amplitudes recorded using K^+^ or NMDG^+^ in the recording electrode solution. Conductance (γ) estimated by fitting the average single channel currents to a linear equation. Error (grey) indicate SEM from K^+^ (N=2–12 GUVs) and NMDG^+^ (N=1–5 GUVs) conditions.

Native and transgene expressed PKD2L1 channels conduct monovalent cations, thus we established symmetric potassium ion (K^+^) recording conditions to measure synthetic polycystin currents from GUVs. GUVs containing PKD2L1 were voltage-clamped using 4–7 MΩ resistance (R) glass electrodes in the inside-out membrane patch configuration (Figure 3A). After obtaining high resistance seals (R >10 GΩ), single channel events were observed in 23% (26/113 GUVs). However, many of these GUV patches were unstable during voltage steps and were excluded from the final analysis. From the stable recordings, two magnitudes of single channels were readily observed from GUVs containing PKD2L1, suggesting unique full and sub-conductance states (Figure 3B, C, Table 1). In some recordings, only one conductance predominates, which can be estimated from recoding from individual GUVs (Figure 3— figure supplement 1A, B). While in other recordings, both full (*FC*) and sub conductive (*SC*) states can be identified by histogram analysis of the unitary current (Figure 3— figure supplement 1C, D). Importantly, no single channel events were observed from GUVs (N= 11) derived from CFE reactions with the empty plasmid— indicating that the measured conductance is not due to a contaminates from lipid or cell-free reagents (Figure 3B). To assess the selectivity of the synthetic PKD2L1 pore, we substituted the pipette K^+^ charge carrier for methyl-D-glucamine ions (NMDG^+^). We did not observe any outward single channel currents (i.e. towards the bath), indicating the large cation was not permeable through PKD2L1 which is consistent with previous reports (Figure 3D, E)^40, 41^. To determine the feasibility of using this approach to assess the function of other polycystin channels, we followed the same steps to assay PKD2 channel biophysics. Single channels were observed more frequently from PKD2 incorporated GUVs (63% or 32/49) compared to PKD2L1 GUVs. As observed with PKD2L1 GUVs, the membrane resistance of synthetic PKD2 GUVs were often destabilized by voltage steps. Importantly, this membrane instability was not observed from empty GUV recordings, suggesting that opening of incorporated polycystin channels during voltage transitions is responsible for patch destabilization. As observed in our PKD2L1 results, unitary single channel currents of synthetic PKD2 channels reconstituted in GUVs yield sub- and full conductances, which were blocked by substitution of K^+^ with NMDG^+^ in the electrode (Figure 4A–C; Figure 3— figure supplement 1E–H). To compare the properties of the synthetic and biologically derived channels, we recorded native PKD2L1 and PKD2 channel single channel currents from the primary cilia membranes of hippocampal neurons and inner medullary collecting duct (IMCD) cell line, respectively (Figure 4—figure supplement 1)^34, 42, 43^. Like the synthetic PKD2 and PKD2L1 channels, native polycystins produced sub and full K^+^ conductances with inward currents having the greater magnitudes. Here, the synthetic PKD2L1 GUV conductance approximates the native full and subconductances recorded from hippocampal primary cilia membranes cultured from neonatal mice (ARL13B-EGFP^tg^). However, the PKD2 K^+^ conductance magnitudes recorded IMCD cilia were significantly larger than those assayed using the CFE-GUV synthetic system (Table 1). These differences might arise from the lack of post-translational modifications (e.g. phosphorylation and N-glycosylation) to the synthetic PKD2 peptides, which are normally found in biologically derived channels^44–46^. In addition, the GUVs are comprised of synthetic lipids which does not reflect the composition of organelle (cilia or ER) membranes of the cell^47^. Thus, while retaining the native ion selectivity and ion channel functionality despite their cell-free origin, synthetic PKD2 has different conductance magnitudes compared to cell derived channels, which presents a limitation of using this approach to recapitulating physiological channel functions.

In summary, we have established a synthetic approach to assay ion channel biophysics using two polycystin members to validate our method. Previously CFE has been used to study membrane protein integration, drug delivery, and the study of actin dynamics^29, 48, 49^. The novelty of our approach rests with the adaptation CFE derived channels and their GUV reconstitution to carry out single channel electrophysiology experiments. The described method represents a highly reductionist approach to assay channel function which can be generalized other channels resistant to characterization using traditional electrophysiology approaches. Furthermore, the CFE-GUV electrophysiology method can be leveraged for future inquiry into lipid-channel regulation; effects of channel subunit composition; and ion channel pharmacology.

## METHODS

### Protein production

Cell-free protein production was performed with PURExpress In Vitro Protein Synthesis Kit from New England Biolabs, Inc. (Ipswich, MA, USA). We utilized the manufacturer’s protocol with 1 mg target DNA and a maximum reaction volume of 30 μl. When appropriate, we added substituted DiH2O for SUVs. The reactions were placed in 37°C water bath or heated plate reader between 2 – 3 hours and then placed in 4°C for storage.

### Vesicle formation

Lipids 1,2-diphytanoyl-sn-glycero-phosphocholine (DPhPC 4ME16:0PC) and cholesterol (ovine) were obtained from Avanti Polar Lipids (Alabaster, AL, USA) and mixed in chloroform to the desired mole percentage, 95% DPhPC and 5% cholesterol. SUVs were formed following the previously described [25]. Briefly, lipids were reconstituted in chloroform in a glass vial and the chloroform was evaporated until a thin lipid layer is deposited on the bottom of the glass vial. The lipid layer is then placed under vacuum, −23 inhg, for >4 hours. Lipids are then rehydrated in 1ml of diH2O overnight at 60°C. The following day, lipids are vortexed and then passed through a 100 nm polycarbonate filter with the Mini-Extruder (Avanti Polar Lipids, Alabaster, AL USA) 7 times and stored at 4°C for two weeks. SUVs with or without channel incorporated are dried onto indium tin oxide coated glass slides from Nanion Technologies (Munich, Germany). The dried slides are placed on the Vesicle Prep Pro (Nanion Technologies) with a rubber o-ring and 300 mM sucrose. GUVs are formed using the standard program. Upon GUV formation, vesicles are used same day.

### Monitoring fluorescence and cell free protein synthesis quantification

We monitored fluorescent folding with PKD2L1 c-terminally tagged GFP during PURExpress reaction in the presence and absence of SUVs. GFP fluorescence was monitored every 10 minutes for three hours at 37°C on the BioTek Cytation5 Imaging Reader (Agilent, Santa Clara, CA, USA). Control plasmid was the PURExpress Control DHFR Plasmid (NEB, Ipswich, MA USA) with no fluorescent tag. Green fluorescent protein (GFP) standard curve was created from dilutions of Aequorea Victoria GFP His-tag recombinant protein (ThermoFisher Scientific, Cat. No. A42613) and measured on the BioTek Cytation5 Imaging Reader. A linear regression (Igor Pro, WaveMetrics, Portland, Or.) was used to create a standard curve. Target protein fluorescent measurements were made after in vitro protein synthesis for three hours.

### Western blot

Western blotting was performed on PKD2L1–GFP plasmid after PURExpress protein expression in the presence of SUVs. SUV and protein mixture were separated on SDS-PAGE gel, Novex Tris-Glycine mini protein gels, 4–20%, 1.0 mm, WedgeWell format, (ThermoFisher, Waltham, Ma USA). The SDS-PAGE was run with 10 μl Spectra Multicolor Broad Range Protein Ladder (ThermoFisher Scientific, Cat. No. 26634). The gel was then transferred to Amersham Hybond P 0.45 PVDF blotting membrane (Cytiva, Cat. No. 10600029) and PKD2L1-GFP was detected with an anti eGFP monoclonal antibody (F56–6A1.2.3) (Invitrogen, Cat. No. MA1–952) diluted 1:1000 in TBS with 0.1% (v/v) Tween-20 and 5% (5/v) milk overnight at 4°C. The goat anti-mouse AF555 secondary (Invitrogen, Cat. No. A32727) diluted 1:5000 in TBS with 0.1% Tween-20 and 5% milk was incubated for 1 hour at room temperature.

### SNAP staining

Channel orientation was visualized with n-terminally tagged PKD2L1 with SNAP Tag sequence, (NEB, Ipswich, MA USA). After PKD2L1-SNAP-tag production and incorporation into GUVs, two SPAP-tag substrates were added to the solution, cell permeable SNAP-Cell Oregon Green and the cell impermeable SNAP-Surface Alexa Fluor 647, according to manufacturer’s instructions. Images were collected on Nikon A1 confocal microscope and all images were analyzed with Nikon Elements (Melville, N USA). Briefly, regions of interests were manually outlined around the vesicle membrane. Then Pearson’s correlation coefficients were measured for fluorescence overlap of the two substrates.

### Mammalian culture

HEK 293T cells were authenticated by supplier, ATCC (Manassas, VA USA) and were transiently transfected with jetPRIME transfection reagent, (Axil Scientific Pte Ltd, Singapore), with mammalian cell expression vector pTracer PKD2L1 with IRES GFP. Transfected cells were seeded onto glass coverslips and placed in a recording chamber for cell-attached electrophysiology recordings.

### Electrophysiology

All research chemicals were purchased for the electrophysiology experiments from Millipore-Sigma. Single-channel recordings were recorded from primary cilia and GUV membranes. All GUV patch electrodes were made using borosilicate glass electrodes and were fire polished to resistances greater than 5 – 10 MΩ and primary cilia patch electrodes were polished to a resistance greater than 18 MΩ. Recording solutions for mammalian culture consisted of symmetrical recording solutions with 150 mM KCl, 10 mM HEPES, and 300 mM glucose, unless the charge carrier was changed when mentioned. Recordings were collected in voltage-clamp with voltage-steps from −100 mV to +100 mV and a holding potential of −40 mV with ClampEx v.11.2.0.59 (MolecularDevices, San Jose, CA USA) using a Axopatch 200B amplifier. Recordings were digitized with the Digidata 1550B (MolecularDevices) at 25kHz and low pass filtered at 5kHz. Recordings were analyzed with ClampFit v11.2.0.59 (Molecular Devices, San Jose, CA USA) and IGOR Pro 8.04 (Wavemetrics, Portland, OR USA). Conductance was determined by determining the slope of the current-voltage relationship. Probability open time was calculated by measuring the time at which a channel spends in an open confirmation divided by the total time in the voltage step.

## Supplementary Material

1

## Figures and Tables

**Table 1.** Conductance properties of polycystins measured from GUV and cilia membranes.

Polycystin channel and membrane context.	Major K^+^ γ (pS) ± S.D.		Minor K^+^ γ (pS) ± S.D.	
	*inward*	*outward*	*inward*	*outward*
PKD2 (GUV membrane, cell free expression)	282 ± 38	153 ± 32	23 ± 4	21 ± 3
PKD2 (primary cilia membrane, endogenous murine IMCD cell line)	144 ± 9	110 ± 6	46 ± 4	34 ± 4
PKD2L1 (GUV membrane, cell free expression)	148 ± 29	114 ± 28	30 ± 6	31 ± 7
PKD2L1 (primary cilia membrane, endogenous hippocampal neurons)	165 ± 10	113 ± 6	40 ± 4	29 ± 3
