# Supplementary material for: A synthetic method to assay polycystin channel biophysics
Source: bioRxiv. 2024 Aug 10:2024.05.06.592666. Originally published 2024 May 6. Preprint. [Version 2] doi: 10.1101/2024.05.06.592666 (PMC11100589; doi:10.1101/2024.05.06.592666)
Supplement: Supplement 1 — Figure 1— figure supplement 1. Quantification, identification and assembly of cell-free synthesized polycystin channels. A). Top, Fluorescence (488μ) standard curve determined with a recombinant GFP tagged protein fit with a linear regression. PURExpress synthesized PKD2L1-GFP and PKD2-GFP were measured after three hours of expression at 37°C. (GFP standard curve N=5, PKD2L1 and PKD2 N=3 replicates). Bottom, average protomer and tetramer protein production from the PURExpress reaction. Error bars represent SEM. B) Representative sequence coverage of PKD2L1-GFP (top) and PKD2-GFP (bottom) from tandem mass spectrometry spectra. C) Mass spectrometry outputs identifying polycystin proteins. D, E) Fluorescence-detection size-exclusion chromatography (FSEC) of polycystin proteins derived from recombinant and CFE sources. Recombinant human PKD2-GFP and PKD2L1-GFP protein was obtained from lysates of 0.5×10^6^ HEK cells stably expressing the channels. Purified Aequorea Victoria GFP His-tag protein was obtained from ThermoFisher Scientific. SUVs containing CFE derived polycystins were lysed using dodecyl β-D-maltoside (DDM) prior to FSEC analysis (see methods). Figure 2— figure supplement 1. The SNAP- fluorescence approach to assess polycystin protein orientation in GUV. Schematic of PKD2L1-SNAP incorporated into GUVs, followed by SNAP-staining with cell permeable (Cell488), and cell impermeable (Surface647) SNAP-Tag marker. Figure 3—figure supplement 1. Synthetic polycystin channels exhibit full and sub-conductive states in GUVs. A, E ) Single channel current amplitudes measured from individual GUVs (open circles). GUVs with open channel events measure at four or more potentials in inward and outward direction were fit to a linear equation to estimate their conductance. B, F) Resulting violin plots of the sub (SC, blue) and full (FC, black) polycystin conductance as estimated from individual GUV recordings (N = 5–7 GUVs). C, G) Unitary single channel currents measured from GU [file media-1.pdf]

A

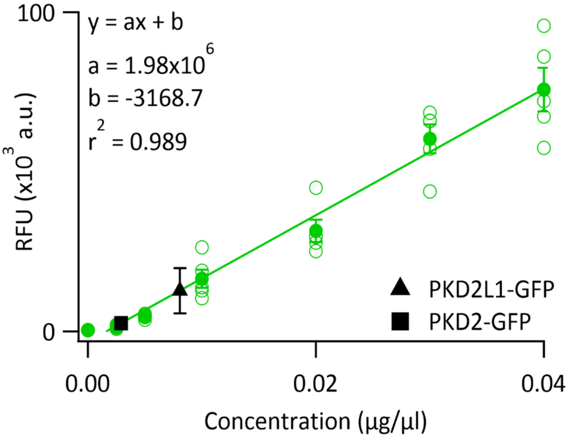

	Average protomer [ng/ul]	Average tetramer [ng/ul]
PKD2L1-GFP	8.0 ± 3.6	2.0 ± 0.9
PKD2-GFP	3.1 ± 2	0.8 ± 0.07

D

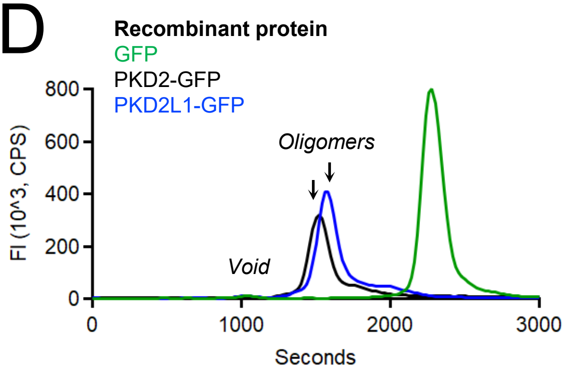

E

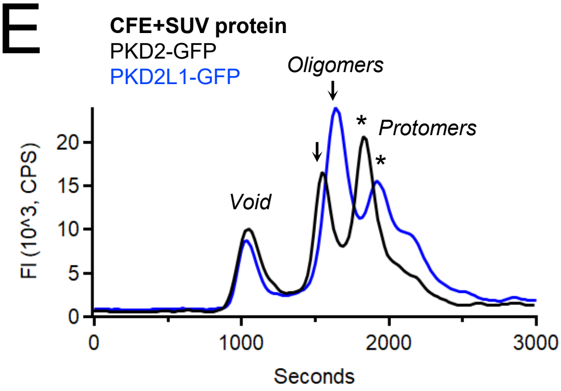

B

Peptide coverage

PKD2L1-GFP

M N A V G S P E G Q E L Q K L G S G A W D N P A Y S G P P S P H G T L R V C T I S
S T G P L Q P Q P K K P E D E P Q E T A Y R T Q V S S C C L H I C Q G I R G L W G T
T L T E N T A E N R E L Y I K T T L R E L L V Y I V F L V D I C L L T Y G M T S S S A Y
Y Y T K V M S E L F L H T P S D T G V S F Q A I S S M A D F W D F A Q G P L L D S L
Y W T K W Y N N Q S L G H G S H S F I Y Y E N M L L G V P R L R Q L K V R N D S C
V V H E D F R E D I L S C Y D V Y S P D K E E Q L P F G P F N G T A W T Y H S Q D E
L G G F S H W G R L T S Y S G G G Y Y L D L P G S R Q G S A E A L R A L Q E G L W
L D R G T R V V F I D F S V Y N A N I N L F C V L R L V V E F P A T G G A I P S W Q I
R T V K L I R Y V S N W D F F I V G C E V I F C V F I F Y Y V V E E I L E L H I H R L R
Y L S S I W N I L D L V V I L L S I V A V G F H I F R T L E V N R L M G K L L Q Q P N T
Y A D F E F L A F W Q T Q Y N N M N A V N L F F A W I K I F K Y I S F N K T M T Q L S
S T L A R C A K D I L G F A V M F F I V F F A Y A Q L G Y L L F G T Q V E N F S T F I
K C I F T Q F R I I L G D F D Y N A I D N A N R I L G P A Y F V T Y V F F V F F V L L N
M F L A I I N D T Y S E V K E E L A G Q K D E L Q L S D L L K Q G Y N K T L L R L R L
R K E R V S D V Q K V L Q G G E Q E I Q F E D F T N T L R E L G H A E H E I T E L T
A T F T K F D R D G N R I L D E K E Q E K M R Q D L E E E R V A L N T E I E K L G R
S I V S S P Q G K S G P E A A R A G G W V S G E E F Y M L T R R V L Q L E T V L E G
V V S Q I D A V G S K L K M L E R K G W L A P S P G V K E Q A I W K H P Q P A P A V
T P D P W G V Q G G Q E S E V P Y K R E E E A L E E R R L S R G E I P T L Q R S M S
K G E E L F T G V V P I L V E L D G D V N G H K F S V S G E G E G D A T Y G K L T L
K F I C T T G K L P V P W P T L V T T F S Y G V Q C F S R Y P D H M K Q H D F F K S
A M P E G Y V Q E R T I F F K D D G N Y K T R A E V K F E G D T L V N R I E L K G I D
F K E D G N I L G H K L E Y N Y N S H N V Y I M A D K Q K N G I K V N F K I R H N I E
D G S V Q L A D H Y Q Q N T P I G D G P V L L P D N H Y L S T Q S A L S K D P N E K
R D H M V L L E F V T A A G I T H G M D E L Y K

PKD2-GFP

M V N S S R V Q P Q Q P G D A K R P P A P R A P D P G R L M A G C A A V G A S L A A P G
G L C E Q R G L E I E M Q R I R Q A A A R D P P A G A A A S P S P P L S S C S R Q A W S R
D N P G F E A E E E E E E V E G E E G G M V V E M D V E W R P G S R R S A A S S A V S S
V G A R S R G L G G Y H G A G H P S G R R R R R E D Q G P P C P S P V G G G D P L H R H
L P L E G Q P P R V A W A E R L V R G L R G L W G T R L M E E S S T N R E K Y L K S V L R
E L V T Y L L F L I V L C I L T Y G M M S S N V Y Y Y T R M M S Q L F L D T P V S K T E K T
N F K T L S S M E D F W K F T E G S L L D G L Y W K M Q P S N Q T E A D N R S F I F Y E N
L L L G V P R I R Q L R V R N G S C S I P Q D L R D E I K E C Y D V Y S V S S E D R A P F G
P R N G T A W I Y T S E K D L N G S S H W G I I A T Y S G A G Y Y L D L S R T R E E T A A Q
V A S L K K N V W L D R G T R A T F I D F S V Y N A N I N L F C V V R L L V E F P A T G G V
I P S W Q F Q P L K L I R Y V T T F D F F L A A C E I I F C F F I F Y Y V V E E I L E I R I H K L
H Y F R S F W N C L D V V I V V L S V V A I G I N I Y R T S N V E V L L Q F L E D Q N T F P N
F E H L A Y W Q I Q F N N I A A V T V F F V W I K L F K F I N F N R T M S Q L S T T M S R C
A K D L F G F A I M F F I I F L A Y A Q L A Y L V F G T Q V D D F S T F Q E C I F T Q F R I I L
G D I N F A E I E E A N R V L G P I Y F T T F V F F M F F I L L N M F L A I I N D T Y S E V K S
D L A Q Q K A E M E L S D L I R K G Y H K A L V K L K L K K N T V D D I S E S L R Q G G G K
L N F D E L R Q D L K G K G H T D A E I E A I F T K Y D Q D G D Q E L T E H E H Q Q M R D
D L E K E R E D L D L D H S S L P R P M S S R S F P R S L D D S E E D D D E D S G H S S R
R R G S I S S G V S Y E E F Q V L V R R V D R M E H S I G S I V S K I D A V I V K L E I M E R
A K L K R R E V L G R L L D G V A E D E R L G R D S E I H R E Q M E R L V R E E L E R W E
S D D A A S Q I S H G L G T P V G L N G Q P R P R S S R P S S S Q S T E G M E G A G G N
G S S N V H V M S K G E E L F T G V V P I L V E L D G D V N G H K F S V S G E G E G D A T
Y G K L T L K F I C T T G K L P V P W P T L V T T F S Y G V Q C F S R Y P D H M K Q H D F
F K S A M P E G Y V Q E R T I F F K D D G N Y K T R A E V K F E G D T L V N R I E L K G I D
F K E D G N I L G H K L E Y N Y N S H N V Y I M A D K Q K N G I K V N F K I R H N I E D G S
V Q L A D H Y Q Q N T P I G D G P V L L P D N H Y L S T Q S A L S K D P N E K R D H M V L
L E F V T A A G I T H G M D E L Y K

C

	Protein ID	Percent coverage	Exclusive unique peptides
PKD2L1-GFP	100%	46%	22
PKD2-GFP	100%	25%	63

Figure 1—Figure Supplement 1

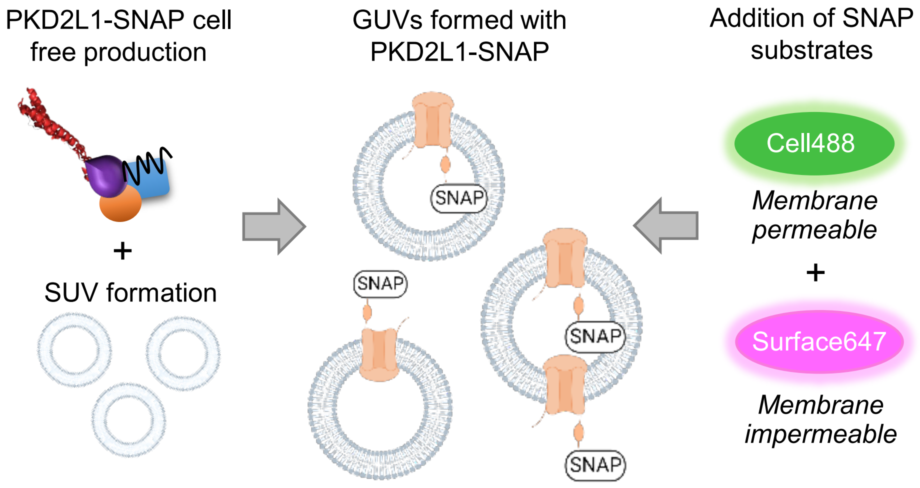

Figure 2– Figure supplement 1

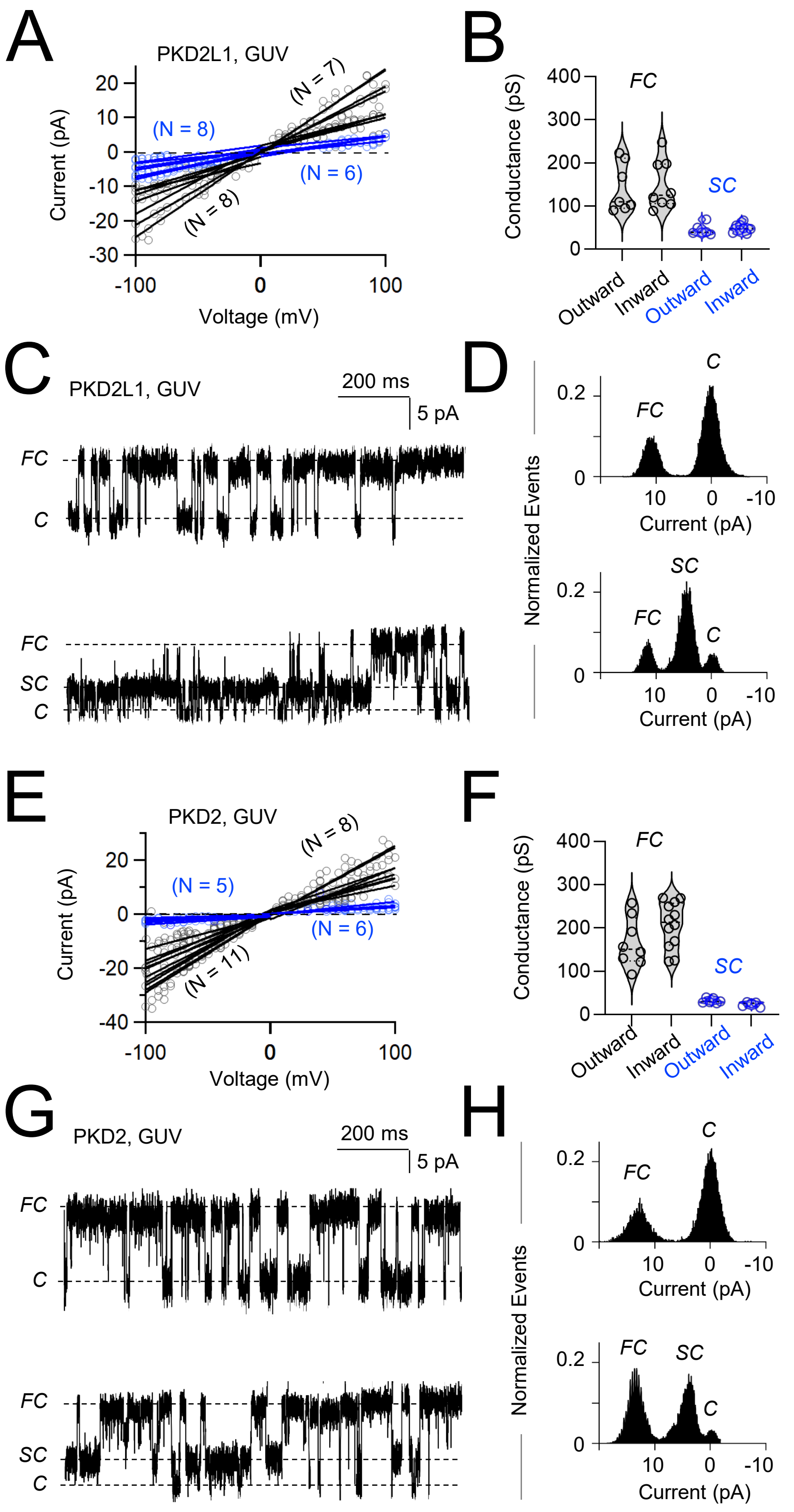

Figure 3– Figure Supplement 1

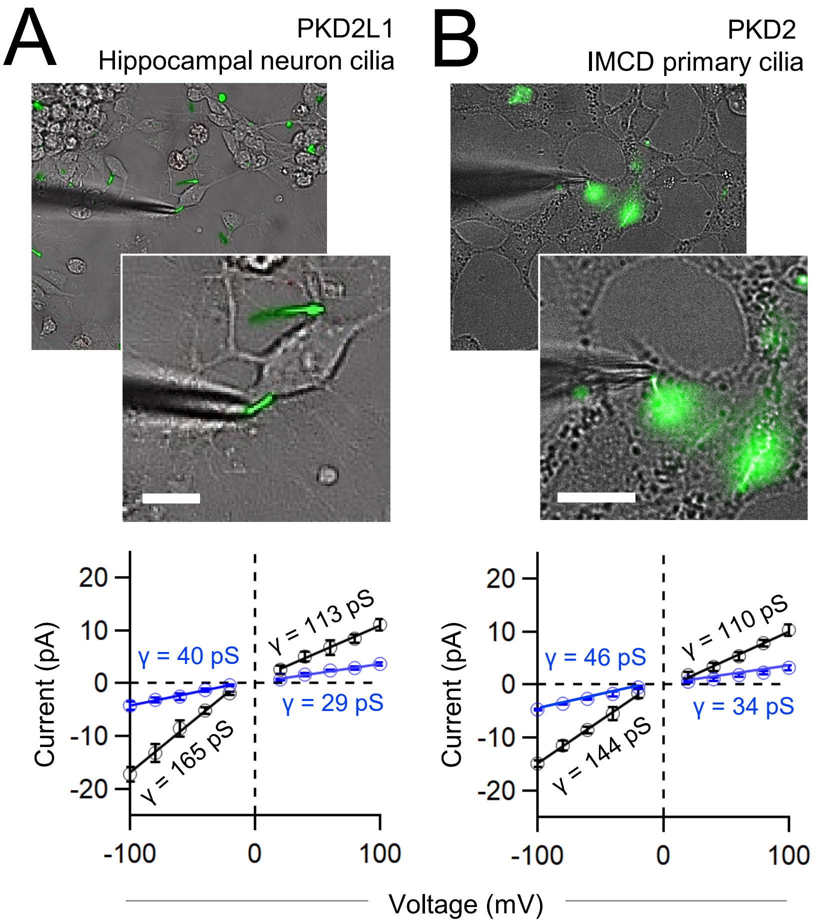

Figure 4– Figure supplement 1
