# Supplementary material for: A synthetic method to assay polycystin channel biophysics
Source: bioRxiv. 2024 May 6:2024.05.06.592666. Preprint. [Version 1] doi: 10.1101/2024.05.06.592666 (PMC11100589; doi:10.1101/2024.05.06.592666)
Supplement: Supplementary file 1 [file NIHPP2024.05.06.592666V1-supplement-1.pdf]

A

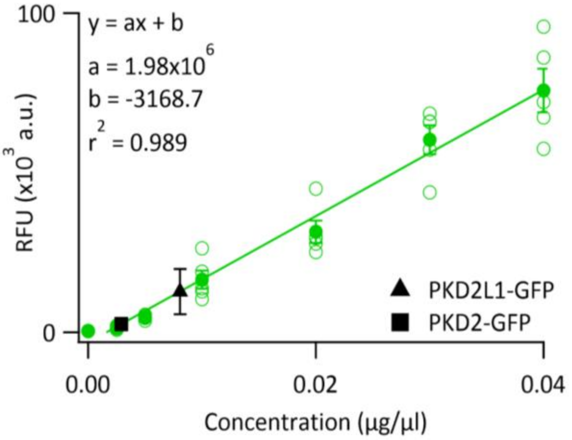

	Average protomer [ng/ul]	Average tetramer [ng/ul]
PKD2L1	8.0 ± 3.6	2.0 ± 0.9
PKD2	3.1 ± 2	0.8 ± 0.07

B

Peptide coverage

PKD2L1-GFP

MNAVGSPEGQELQKLGSGAWDNPAYSGPPSPHGTLRVCTISSTGPLQPQPKKPEDEPQETAYRTQVSSCCLHICQGIRGLW
GTTLTENTAENRELYIKTTLRELLVYIVFLVDICLLTYGMTSSSAYYYTKVMSELFLHTPSDTGVSFQAISSMADFWDFAQGPL
LDSLYWTKWYNNQSLGHGSHSFIYYENMLLGVPRLRQLKVRNDSCVVHEDFREDILSCYDVYSPDKEEQLPFGPFNGTAWT
YHSQDELGGFSHWGRLTSYSGGGYYLDLPGSRQGSAEALRALQEGLWLDRGTRVVFIDFSVYNANINLFCVLRLVVEFPATG
GAIPSWQIRTVKLIRYVSNWDFFIVGCEVIFCVFIFYYVVEEILELHIHRLRYLSSIWNILDLVVILLSIVAVGFHIFRTLEVNRL
MGKLLQQPNTYADFEFLAFWQTQYNNMNAVNLFFAWIKIFKYISFNKTMTQLSSTLARCAKDILGFAVMFFIVFFAYAQLGYL
LFGTQVENFSTFIKCIFTQFRIILGDFDYNAIDNANRILGPAYFVTYVFFVFFVLLNMFLAIINDTYSEVKEELAGQKDELQLSD
LLKQGYNKTLLRLRLRKERVSDVQKVLQGGEQEIQFEDFTNTLRELGHAEHEITELTATFTKFDRDGNRILDEKEQEKMRQDL
EEERVALNTEIEKLGRSIVSSPQGKSGPEAARAGGWVSGEEFYMLTRRVLQLETVLEGVVSQIDAVGSKLKMLERKGWLAPS
PGVKEQAIWKHPQPAPAVTPDPWGVQGGQESEVPYKREEEALEERRLSRGEIPTLQRSMSKGEELFTGVVPILVELDGDVN
GHKFSVSGEGEGDATYGKLTLKFICTTGKLPVPWPTLVTTFSYGVQCFSRYPDHMKQHDFFKSAMPEGYVQERTIFFKDDGN
YKTRAEVKFEGDTLVNRIELKGIDFKEDGNILGHKLEYNYNSHNVYIMADKQKNGIKVNFKIRHNIEDGSVQLADHYQQNTPI
GDGPVLLPDNHYLSTQSALSKDPNEKRDHMVLLEFVTAAGITHGMDELYK

PKD2-GFP

MVNSSRVQPQQPGDAKRPPAPRAPDPGRLMAGCAAVGASLAAPGGLCEQRGLEIEMQRIRQAAARDPPAGAAASPSPPLSSCSRQAW
SRDNPGFEAEEEEEEVEGEEGGMVVEMDVEWRPGSRRSAASSAVSSVGARSRGLGGYHGAGHPSGRRRRREDQGPPCPSPVGGGD
PLHRHLPLEGQPPRVAWAERLVRGLRGLWGTRLMEESSTNREKYLKSVLRELVTYLLFLIVLCILTYGMMSSNVYYYTRMMSQLFLDTP
VSKTEKTNFKTLSSMEDFWKFTEGSLLDGLYWKMQPSNQTEADNRSFIFYENLLLGVPRIRQLRVRNGSCSIPQDLRDEIKECYDVYSV
SSEDRAPFGPRNGTAWIYTSEKDLNGSSHWGIIATYSGAGYYLDLSRTREETAAQVASLKKNVWLDRGTRATFIDFSVYNANINLFCVV
RLLVEFPATGGVIPSWQFQPLKLIRYVTTFDFFLAACEIIFCFFIFYYVVEEILEIRIHKLHYFRSFWNCLDVVIVVLSVVAIGINIYRTSNVE
VLLQFLEDQNTFPNFEHLAYWQIQFNNIAAVTVFFVWIKLFKFINFNRTMSQLSTTMSRCAKDLFGFAIMFFIIFLAYAQLAYLVFGTQVD
DFSTFQECIFTQFRIILGDINFAEIEEANRVLGPIYFTTFVFFMFFILLNMFLAIINDTYSEVKSDLAQQKAEMELSDLIRKGYHKALVKLKL
KKNTVDDISESLRQGGGKLNFDELRQDLKGKGHTDAEIEAIFTKYDQDGDQELTEHEHQQMRDDLEKEREDLDLDHSSLPRPMSSRSF
PRSLDDSEEDDDEDSGHSSRRRGSISSGVSYEEFQVLVRRVDRMEHSIGSIVSKIDAVIVKLEIMERAKLKRREVLGRLLDGVAEDERL
GRDSEIHREQMERLVREELERWESDDAASQISHGLGTPVGLNGQPRPRSSRPSSSQSTEGMEGAGGNGSSNVHVMSKGEELFTGVVP
ILVELDGDVNGHKFSVSGEGEGDATYGKLTLKFICTTGKLPVPWPTLVTTFSYGVQCFSRYPDHMKQHDFFKSAMPEGYVQERTIFFKD
DGNYKTRAEVKFEGDTLVNRIELKGIDFKEDGNILGHKLEYNYNSHNVYIMADKQKNGIKVNFKIRHNIEDGSVQLADHYQQNTPIGDGP
VLLPDNHYLSTQSALSKDPNEKRDHMVLLEFVTAAGITHGMDELYK

C

	Protein identification probability	Percent coverage	Exclusive unique peptides
PKD2L1-GFP	100%	46%	22
PKD2-GFP	100%	25%	63

Figure 1—Figure Supplement 1

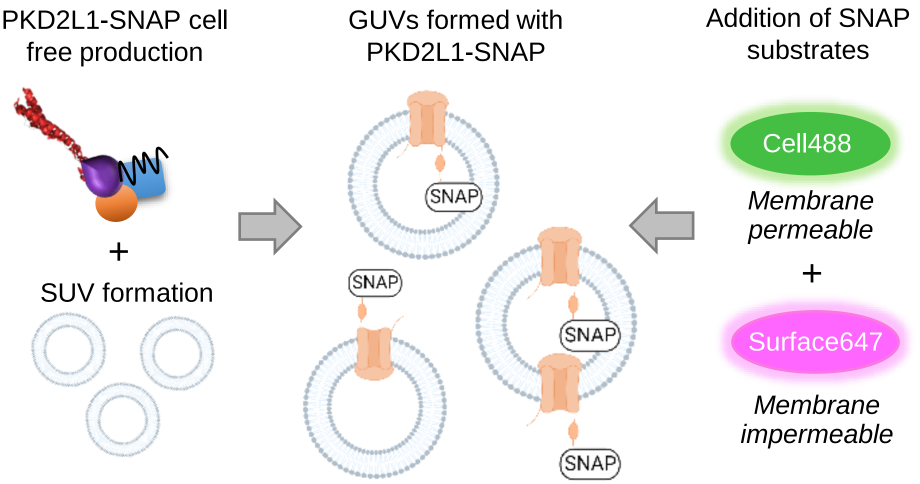

Figure 2– Figure supplement 1

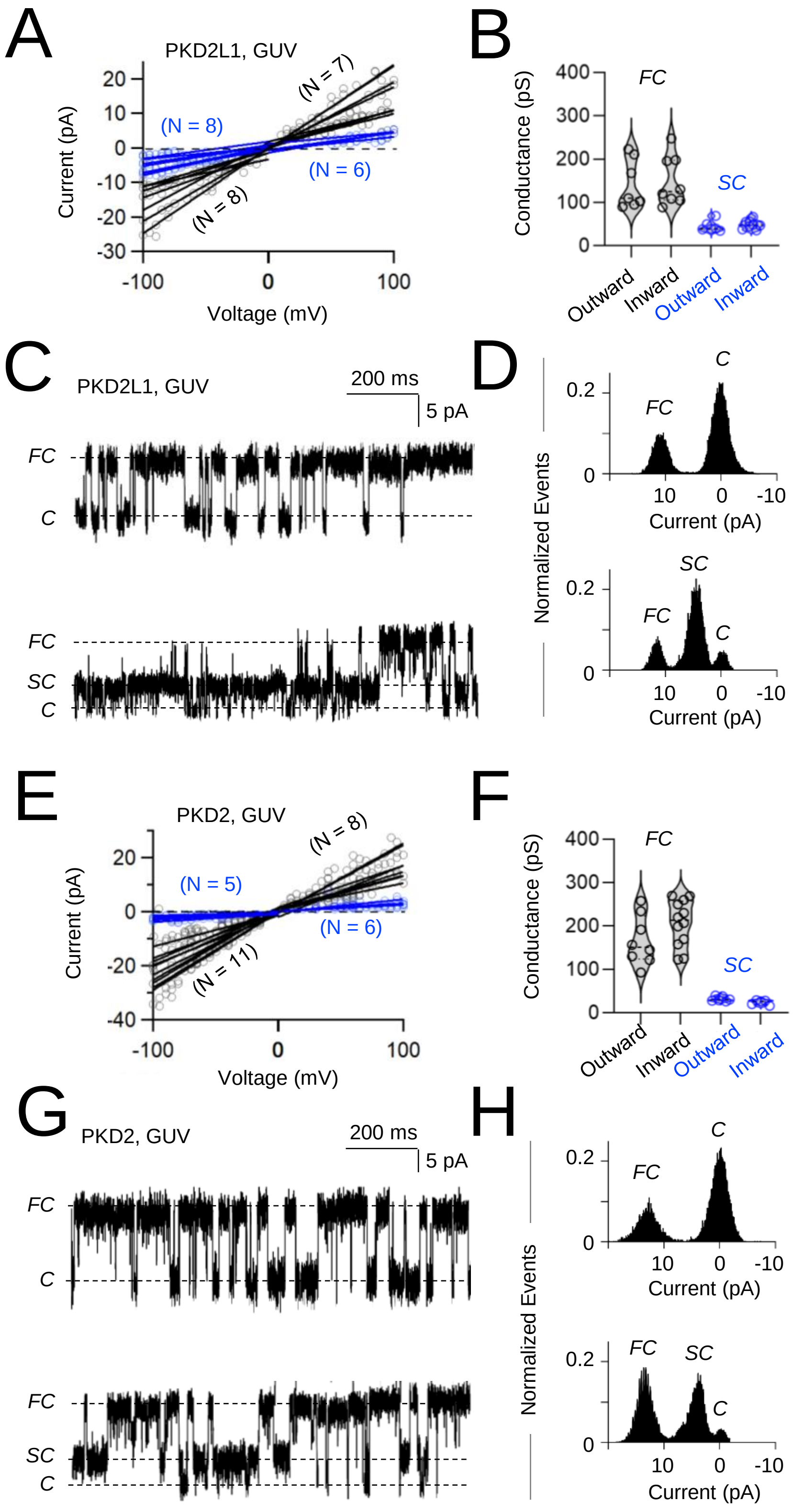

Figure 3– Figure Supplement 1

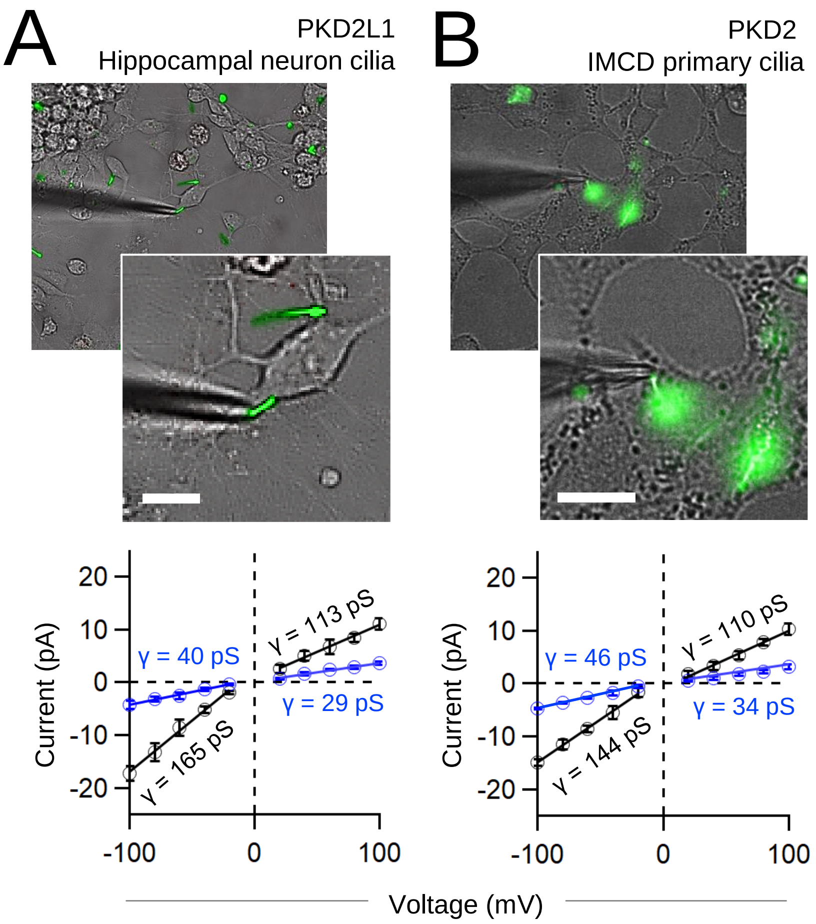

Figure 4– Figure supplement 1
